# Improved Method for the Calculation of the Air Film Thickness of an Air Cushion Belt Conveyor

**DOI:** 10.3390/ma17236020

**Published:** 2024-12-09

**Authors:** Bo Song, Hongliang Chen, Long Sun, Kunpeng Xu, Xiaoyong Ren

**Affiliations:** 1 Digital and Intelligent Industry Center, CCTEG Shenyang Engineering Company, Shenyang 110013, China15502498996@163.com (L.S.); 2 School of Mechanical Engineering and Automation, Northeastern University, Shenyang 110013, China; 3 School of Mechanical and Electrical Engineering, China University of Mining & Technology (Beijing), Beijing 100083, China

**Keywords:** air cushion belt conveyor, air film thickness, calculating method, fluid simulation

## Abstract

The air film thickness is an important parameter of an air cushion belt conveyor, which directly affects the compressed air supply power and operating resistance of the system. Therefore, it is important to calculate the bottom thickness of the gas film accurately in the design stage. A calculation method for the thickness of a conveyor air cushion was derived based on the mathematical model of the air cushion flow field for a multi row uniformly distributed air cushion structure. Meanwhile, the algorithm was validated based on a Fluent 3D flow field numerical simulation and experiments. Through verification, it was found that due to the algorithm’s assumption that the increase in the gas flow rate only existed at the axis of the gas hole, there was a sudden change in the calculation results of the gas flow rate at the axis of the gas hole. The sudden change in the gas flow rate had caused the calculation results of the air cushion thickness to experience abrupt and discontinuous changes. Furthermore, the calculation method for air cushion thickness was revised based on the verification results. Compared with the experimental test results, the average error of the calculation results of the algorithm proposed in this paper was 14.27%.

## 1. Introduction

As important equipment for the continuous transportation of bulk materials, an air cushion belt conveyor has been widely used in ports, grain warehouses, coal mines, and other fields [1,2,3,4,5]. Compared to traditional belt conveyors (using rollers to support the conveyor belt), it can effectively reduce the running resistance and the required driving power of the roller. In addition, it has many advantages such as stable operation, low energy consumption, and low noise [6,7,8,9]. Air film thickness is an important parameter of the air cushion belt conveyor, which directly affects the air supply power and running resistance of the system. Therefore, it has become an important task in the design stage on how to accurately calculate air film thickness. Some scholars have focused on the calculation method of air film thickness and have carried out research. Guo et al. [10] analyzed the air cushion field through theoretical derivation, numerical simulation, and experimental research, developing an intelligent experimental platform. We obtained the three-dimensional pressure distribution and thickness distribution of the gas film along the width direction of the conveyor belt. Fang Yuefeng [11] analyzed the mechanism of air film formation and its influencing factors, and introduced the relationship between the various factors and the way to reasonably select the various parameters. Yu Fang et al. [12] proposed a method for calculating air film thickness, which provided a reliable basis for the design of air cushion belt conveyors. Pang Mingjun [13] introduced four methods for calculating the air film thickness of air cushion belt conveyors based on the theory of incompressible viscous fluid flow, and compared and analyzed the calculation results of the four methods. Zhu Xiaolong et al. [14] constructed a mathematical model of the air film and used the gap flow theory and experimental tests to analyze and study the air film thickness of the air cushion belt conveyor. Zhang et al. [15] established a mathematical model of an air cushion belt conveyor through mechanical analysis, and obtained the relationship between the load distribution, the film thickness, the film carrying capacity, and power consumption. Numerical simulations were conducted on the holes of different diameters on the disk groove and formed air film. By observing the stress and deformation of the steel wire rope core, the bearing capacity of the air film was analyzed. Wang et al. [16] established a mathematical model of an air cushion based on fluid lubrication theory. Using Fluent 2019, the effects of the air cushion thickness, the pore velocity, and the belt velocity on the pressure and bearing characteristics of the air cushion flow field were studied. Zhang et al. [17] used single factor and orthogonal experimental methods to study the influence of the structure and operational factors of the disk groove on the gas film loading performance. In previous studies, scholars have established the air cushion flow field model of the air cushion conveyor and carried out corresponding mechanical analysis. However, few studies have focused on the calculation method of air cushion thickness. Moreover, some calculation methods assume that the increase in pore flow only exists at the hole axis, so the result of the sudden change in flow at the hole axis was obtained in the calculation process, which led to the problem of the sudden discontinuity of the calculation results of the air cushion thickness.

In response to the above issues, in this paper, a calculation method for the air cushion thickness of a belt conveyor with multiple rows of uniformly distributed hole air cushions was derived based on a mathematical model of the air cushion flow field. The validation of the effectiveness of the method was performed through numerical and experimental methods. Based on the verification results, the calculation method for the air cushion thickness has been improved, addressing the issues of sudden and discontinuous results in air cushion thickness calculation.

## 2. Calculation Method of Air Cushion Thickness

**Figure 1:**
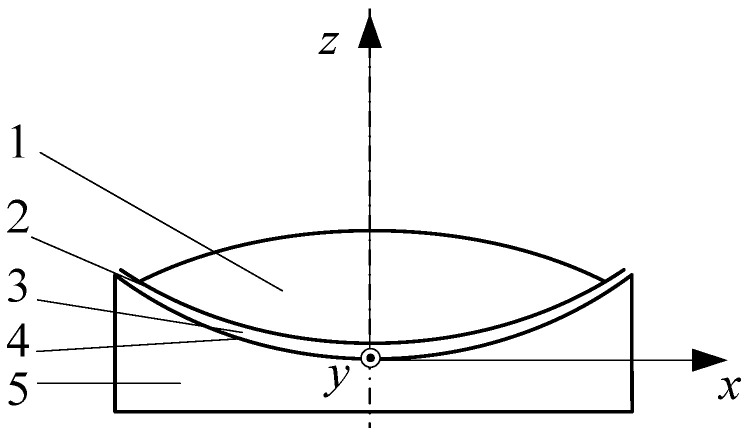
Schematic cross-sectional view of air cushion belt conveyor. 1—material. 2—conveyor belt. 3—air cushion. 4—tray slot. 5—air chamber.

As shown in Figure 1, during the stable operation of the air cushion belt conveyor, the gas flowing through the air holes will form an air cushion with a certain pressure between the disk groove and the conveyor belt. From a geometric perspective, the width of the air cushion flow field in the *x* direction depends on the bandwidth and disk slot structure. The length in the *y* direction is mainly limited by the arrangement length of the disk groove and conveyor belt in the *y* direction. The thickness in the *z* direction is usually small, and the minimum air cushion thickness exists at the edge of the conveyor belt. The scale of the air cushion flow field in length (*y* direction) and width (*x* direction) mainly depends on the structural dimensions of the tray slot and the conveyor belt. Combined with the principle of the air cushion belt conveyor, the size of the air cushion in the thickness direction should be determined by the result of the pore pressure and load pressure. Therefore, the thickness of the air cushion should be reasonably calculated before analysis.

### 2.1. Mathematical Model of Air Cushion Flow Field

In fluid mechanics, the mathematical description of the three conservation laws is shown in Equation (1).

(1) $$\left.\begin{array}{c} \frac{\partial \rho }{\partial t}+\frac{\partial \left(\rho u\right)}{\partial x}+\frac{\partial \left(\rho v\right)}{\partial y}+\frac{\partial \left(\rho w\right)}{\partial z}=0\\ \rho \left(\frac{\partial u}{\partial t}+u\frac{\partial u}{\partial x}+v\frac{\partial u}{\partial y}+w\frac{\partial u}{\partial z}\right)=\rho f_{x}+\frac{\partial p_{xx}}{\partial x}+\frac{\partial \tau_{xy}}{\partial y}+\frac{\partial \tau_{xz}}{\partial z}\\ \rho \left(\frac{\partial v}{\partial t}+u\frac{\partial v}{\partial x}+v\frac{\partial v}{\partial y}+w\frac{\partial v}{\partial z}\right)=\rho f_{y}+\frac{\partial \tau_{yx}}{\partial x}+\frac{\partial p_{yy}}{\partial y}+\frac{\partial \tau_{yz}}{\partial z}\\ \rho \left(\frac{\partial w}{\partial t}+u\frac{\partial w}{\partial x}+v\frac{\partial w}{\partial y}+w\frac{\partial w}{\partial z}\right)=\rho f_{z}+\frac{\partial \tau_{zx}}{\partial x}+\frac{\partial \tau_{zy}}{\partial y}+\frac{\partial p_{zz}}{\partial z}\\ \rho \frac{De}{Dt}=\nabla •\left(\kappa \nabla T\right)-p\nabla •v+\Phi \end{array}\right\}$$

where *ρ* is the fluid density; *u*, *v*, *w* are the components of the fluid velocity ***v*** in the *x*, *y*, and *z* directions, respectively; *t* is the time; *f_x_*, *f_y_*, and *f_z_* are the components of the volume force on the fluid in the *x*, *y*, and *z* directions, respectively; *p* is the thermodynamic pressure; *μ* is the dynamic viscosity of the fluid; *e* is the internal energy per unit mass of the fluid; *κ* is the thermal conductivity; *T* is the thermodynamic temperature; and *Φ* is the viscosity dissipation function.

However, for the air cushion belt conveyor, some physical quantities in the equations cannot be accurately described, so it is very difficult to solve the equations. Therefore, it is necessary to make reasonable assumptions, so as to give the expressions of these physical quantities and simplify the equation.

After forming an air cushion between the conveyor belt and the disk groove, the gas temperature inside the flow field is almost the same as the outside air temperature, and the heat exchange between the air cushion flow field and the outside is extremely small, which can be ignored. In addition, assuming that the material is evenly distributed in the longitudinal direction (*y* direction) of the conveyor belt, it can be simplified as a two-dimensional flow field during static support. Equation (1) can be simplified as

(2) $$\left.\begin{array}{c} \frac{\partial \rho }{\partial t}+\frac{\partial \left(\rho u\right)}{\partial x}+\frac{\partial \left(\rho w\right)}{\partial z}=0\\ \rho \left(\frac{\partial u}{\partial t}+u\frac{\partial u}{\partial x}+w\frac{\partial u}{\partial z}\right)=\rho f_{x}+\frac{\partial p_{xx}}{\partial x}+\frac{\partial \tau_{xz}}{\partial z}\\ \rho \left(\frac{\partial w}{\partial t}+u\frac{\partial w}{\partial x}+w\frac{\partial w}{\partial z}\right)=\rho f_{z}+\frac{\partial \tau_{zx}}{\partial x}+\frac{\partial p_{zz}}{\partial z} \end{array}\right\}$$

Furthermore, according to the actual working state of the air cushion belt conveyor, the thickness of the air cushion is usually in the millimeter order; that is, the scale in the length and width directions is 10^−3^–10^−4^ times the thickness of the air cushion. Therefore, the curvature of the tray slot can be ignored, and the interface between the air cushion flow field and the tray slot can be considered to be a plane. Taking into account the gas flow velocity in the air cushion flow field, the gas flow can be considered to be an incompressible flow. If the load is evenly distributed in the *y* direction, the air supply parameters do not change with the position in the *y* direction, and the two do not change with time. Equation (2) can be further simplified as

(3) $$\frac{\partial p}{\partial x}=\mu \frac{\partial^{2}u}{\partial z^{2}}$$

(4) $$\frac{\partial p}{\partial z}=-\rho g$$

### 2.2. Calculation of Air Cushion Thickness

According to the simplified air cushion flow field mathematical model established in Section 2.1, integrating both sides of Equation (3) with respect to *z* can obtain

(5) $$u=\frac{1}{2\mu }\frac{\partial p}{\partial x}z^{2}+Az+B$$

wherein the constants *A* and *B* can be determined by boundary conditions.

According to the coordinate system shown in Figure 1, the thickness of the air cushion was set to be *h*. When *z* = 0 and *z* = *h*, they represent the contact surface between the air cushion and the conveyor belt and the tray slot, respectively. The speed of the conveyor belt and the tray slot in the *x* direction is 0. Therefore, according to the no-slip condition, the velocity component of the gas at this position *u* = 0. Substituting it into Equation (5), we can obtain

(6) $$u=\frac{1}{2\mu }\frac{\partial p}{\partial x}\left(z^{2}-hz\right)$$

Integrating both sides of Equation (6) from *z* = 0 to *z* = *h* yields

(7) $$h=\sqrt[{3}]{-\frac{12\mu Q}{\partial p/\partial x}}$$

where *Q* is the area flow rate in the *x* direction, that is, the flow rate per unit length of the air cushion m^2^/s.

It can be seen from Equation (7) that the air cushion thickness *h* at a certain position in the air cushion flow field depends on the gas flow rate and the pressure *p* generated by the weight of the material and the weight of the conveyor belt acting on the belt area.

In theoretical analysis, the air cushion pressure *p* can be considered equal to the pressure generated by the conveyor belt and the material gravity; that is

(8) $$\begin{array}{c} p=\frac{G_{BM}}{B}\left(2\cos\left(\sin^{-1}\left(\frac{x}{R}\right)\right)-\cos\lambda \right)+\gamma gR\left(\cos\left(\sin^{-1}\left(\frac{x}{R}\right)\right)-\cos\lambda \right)\\ +R\frac{\sin\lambda }{\sin\beta }\left\{\cos\left(\sin^{-1}\left(\frac{\sin\beta }{\sin\lambda }\frac{x}{R}\right)\right)-\cos\beta \right\} \end{array}$$

wherein *G_BM_* is the weight of the conveyor belt per meter length, N/m; *B* is the width of the conveyor belt, m; *R* is the radius of the circular arc segment of the tray slot, m; *λ* is the maximum tray slot position angle, rad; *γ* is the material density, kg/m^3^; *g* is the acceleration of gravity, m/s^2^; *β* is the material stacking angle, rad.

**Figure 2:**
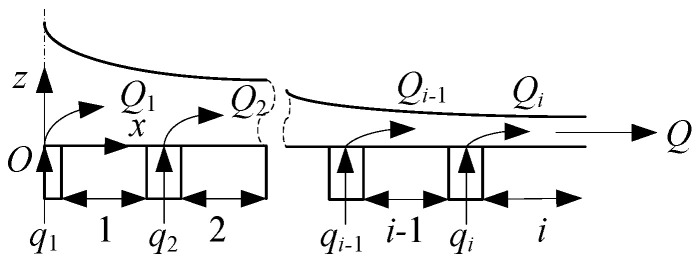
Schematic diagram of air cushion flow field zoning.

The air cushion flow field is divided into *i* intervals in the horizontal direction according to Figure 2, and the gas flow rate *Q* can be calculated according to the following equation:

(9) $$Q=Q_{i}=\sum_{i=1}^{I}q_{i}=\sum_{i=1}^{I}CN_{i}A_{i}\sqrt{\frac{2\left(p_{k}-p_{i}\right)}{\rho }}$$

where *Q_i_* is the flow rate of the *i*-th interval segment, *q_i_* is the gas flow rate of the *i*-th exhaust hole, *C* is the flow coefficient, *N_i_* is the number of air holes opened per meter length in the *i*-th row, *A_i_* is the area of a single air hole in the *i*-th row, *p_k_* is the chamber pressure, and *p_i_* is the air cushion pressure at the corresponding position of the air hole.

Combined with Equations (6)–(8), the lateral distribution of the air cushion thickness can be calculated when the air cushion thickness is known at one point.

## 3. Verification of Air Cushion Thickness Calculation Method

In order to verify the above calculation method of the air cushion thickness, two verification routes are formulated in this thesis:

(1) Based on the calculation results, Fluent is used to simulate the air cushion flow field in three dimensions, and the transverse distribution of the air cushion pressure from the simulation results is extracted. The authenticity of the proposed method is verified by comparing the pressure distributions of the two methods;

(2) By comparing with the experimental measurement results, the authenticity of the proposed method is verified, known as experimental verification.

**Figure 3:**
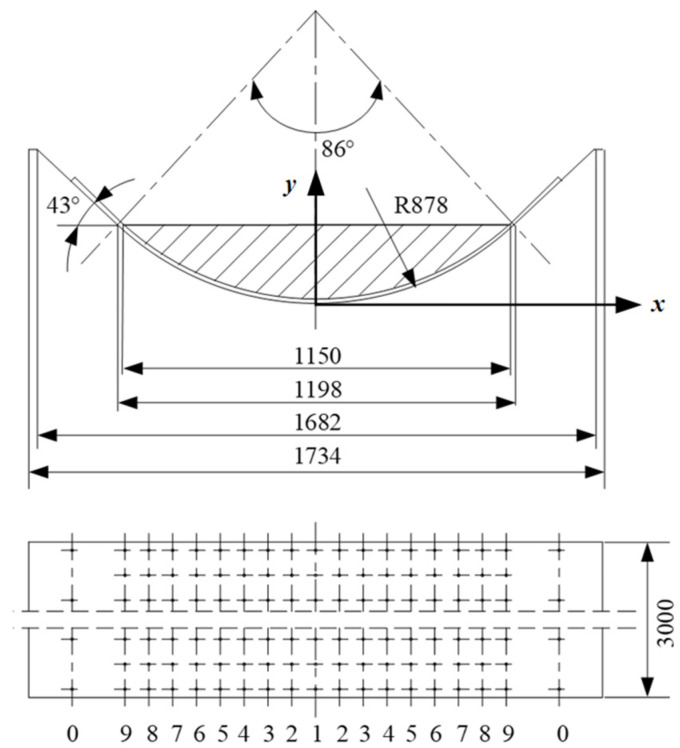
Schematic diagram of the structure of the test tray slot.

Next, simulation calculations and experimental tests will be conducted on the following test bench structure. The specific structure and parameters of the test bench are as follows:

The tray slot structure and parameters used in the test are shown in Figure 3. The length of a single tray slot is 3 m, and the total length of the layout during the test is 9 m. The 1st to 9th rows of holes are arranged with equal row spacing and equal hole spacing, but an unequal hole diameter. The row spacing is 0.072 m, the hole spacing is 0.075 m, and the hole diameters of each row are shown in Table 1. The 0th row of holes is a correction hole, and the distance from the 9th row is 0.16 m. The conveyor belt model is HHE3500, the belt width is 1.8 m, and the conveyor belt mass is 405 kg. The test material is sand, which is evenly arranged on the conveyor belt. The stacking area is shown in the shaded part of Figure 3. The material stacking angle is 0°, the chord length is 1.15 m, and the total mass is about 2000 kg. The fan model is 9–19 No.4.75 A, the air volume *Q* = 1702 m^3^/h, the output pressure *p* = 5552 Pa, the speed *n* = 2900 r/min, and the power *N* = 7.5 kW.

### 3.1. Numerical Validation

**Figure 4:**
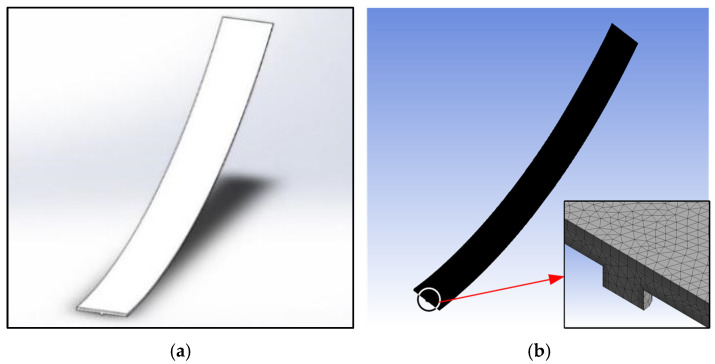
Three-dimensional model of air cushion flow field. (**a**) Geometric model; (**b**) Mesh model.

The software used for air cushion flow field simulation is Fluent [18]. When establishing the 3D model, a spline curve is established as the interface between the air cushion and the conveyor belt based on the thickness of the air cushion at each exhaust hole position in the air cushion thickness calculation result, and then the interface between the air cushion and the conveyor belt is generated. The 3D model is shown in Figure 4, which intercepts the air cushion flow field along the longitudinal length of the tray slot 75 mm and the transverse position of the tray slot 0≤x≤575. This section of the flow field area is used to approximate the section where the air cushion carries both the conveyor belt and the material. The geometric parameters of the interface between the air cushion flow field and the tray slot depend on the structural dimensions of the tray slot and the pores. When dividing the mesh, the maximum mesh size is limited to 1 mm, and the boundary layer mesh is divided at the interface between the tray slot and the air cushion, the interface between the conveyor belt and the air cushion, and the surface of the pore wall. The completely meshed model has about 1,300,000 elements. The solver and boundary condition settings are shown in Table 2.

According to the calculation method, 15 groups of air cushion thickness values were calculated, and the flow field numerical simulation was performed one by one using Fluent. The magnitude of the vertical force (*y* direction) on the interface between the air cushion and the conveyor belt in the simulation results was extracted and compared with the sum of the gravity of the conveyor belt and the material (about 93.9 N).

### 3.2. Test Verification

**Figure 5:**
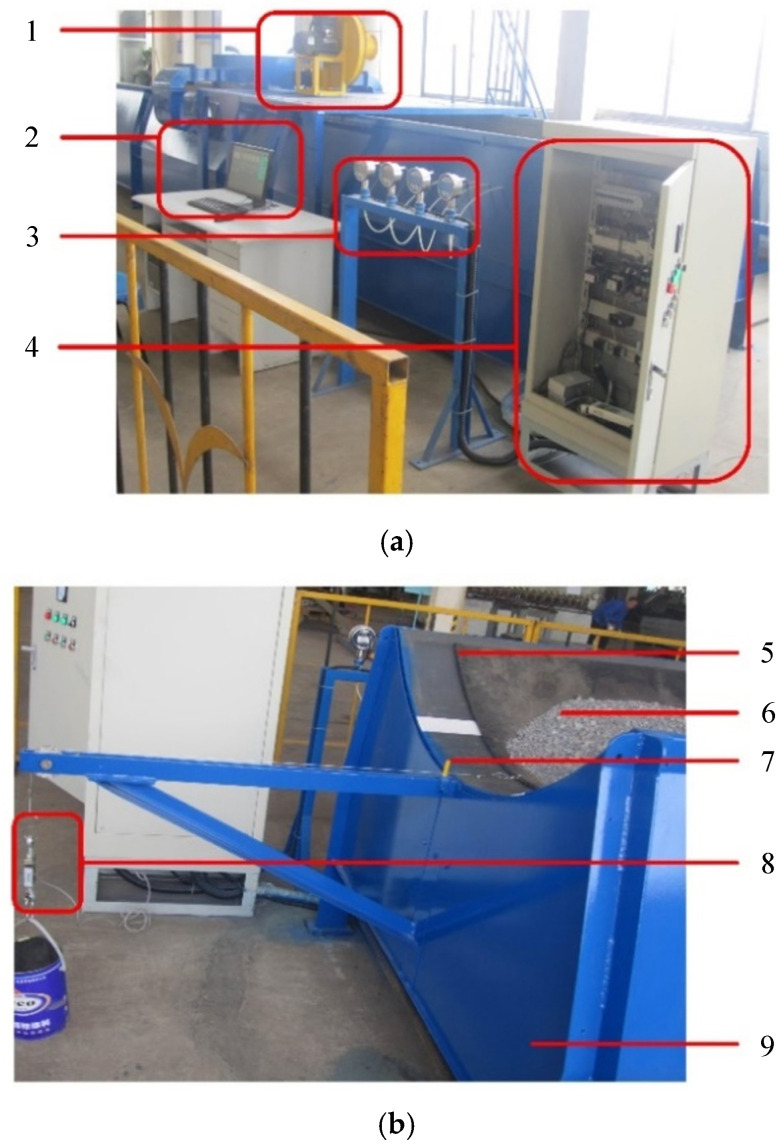
Schematic diagram of test site. 1—centrifugal high pressure fan. 2—control and data comprehensive display system. 3—digital pressure gauge. 4—central control electrical cabinet. 5—conveyor belt. 6—material. 7—tray slot. 8—digital tension meter. 9—air chamber. (**a**) Control and testing equipment; (**b**) Air cushion conveyor.

The test site is shown in Figure 5. In the experiment, the pressure of the air cushion and the air chamber and the thickness of air cushion were measured using a digital pressure gauge and the percentage rule, respectively, and the measured data were transmitted to the corresponding interface in the central control electrical cabinet, and collected and processed in the data comprehensive display system in the computer wherein a percentage rule for measuring the thickness of the air cushion was arranged on the upper surface of the material. The digital tensile meter shown in Figure 5b was used to measure the tensile force of the conveyor belt, and it was also used to match the weight in the test to give the initial tensile force of the conveyor belt.

During the test, the running speed of the conveyor belt was 0, and the air supply parameters were changed by changing the fan gear. The air chamber pressure under each the air supply parameter and the air cushion thickness at five different positions were measured. The test results are shown in Table 3.

According to the test situation, the air cushion pressure value and pressure distribution of the section carrying the material and the conveyor belt at the same time in the transverse direction can be calculated by Equation (8). The parameter values involved in the equation are shown in Table 4.

**Figure 6:**
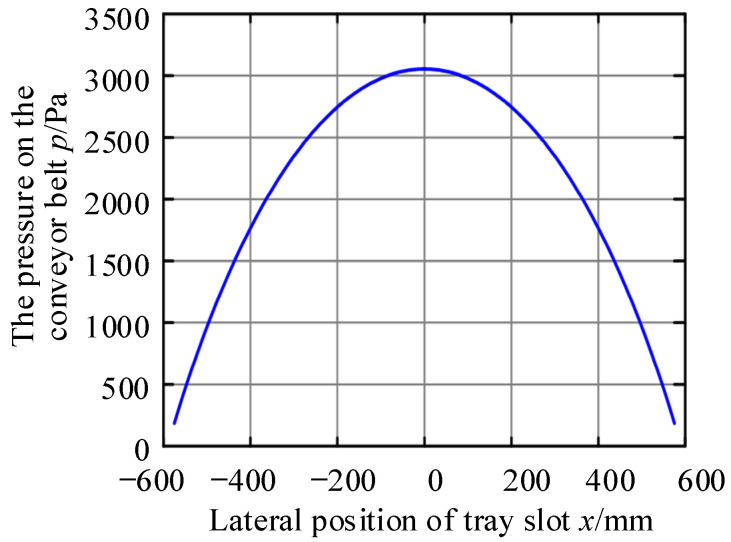
Theoretical distribution of air cushion pressure.

According to the parameters listed in the table, combined with Figure 3 and substituted into Equation (8), the theoretical pressure values of each position in the air cushion area can be calculated, and the results are shown in Figure 6.

**Figure 7:**
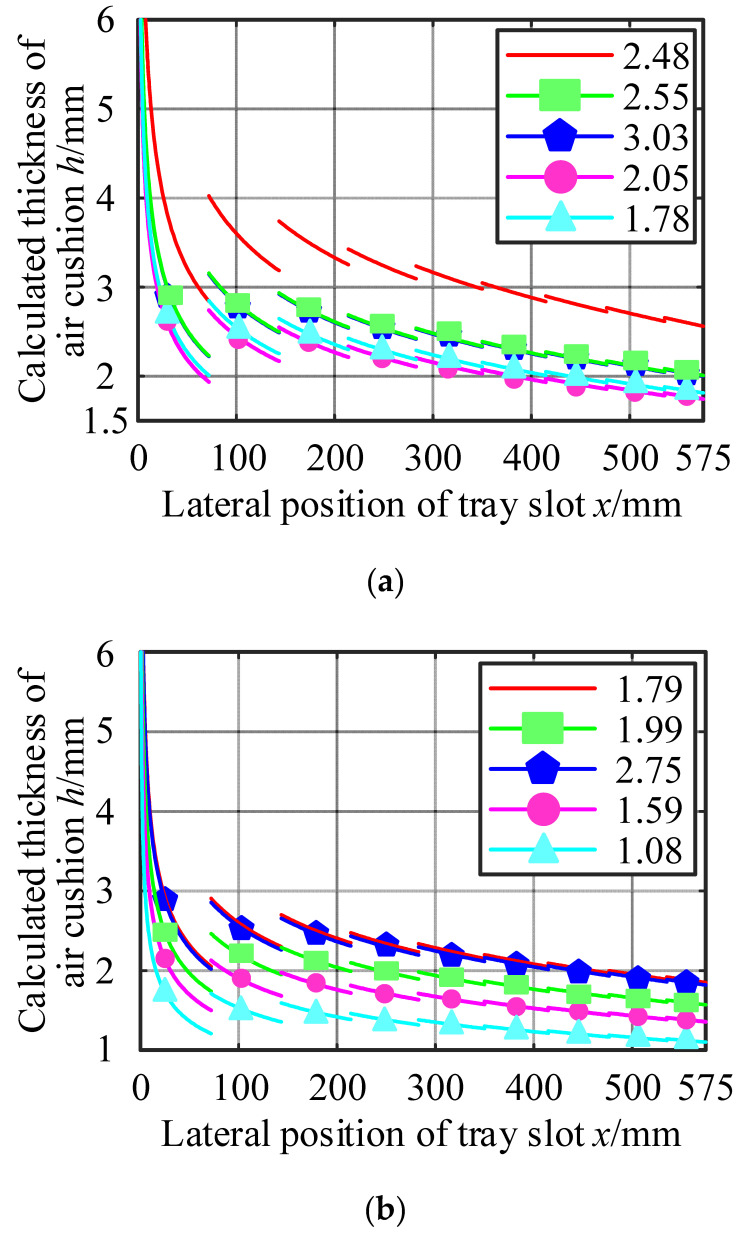

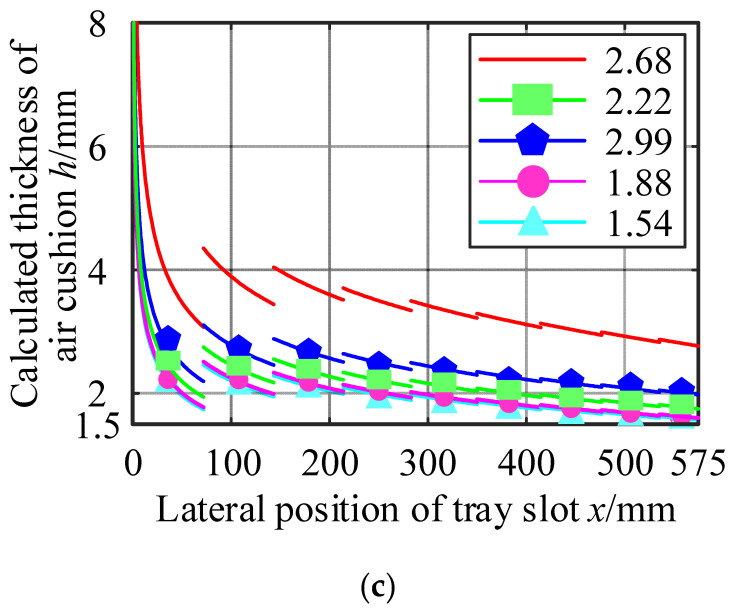
Air cushion thickness distribution curve. (**a**) Substitute Experiment 1 data; (**b**) substitution of Experiment 2 data; (**c**) substitution of Experiment 3 data.

Taking the test results as known quantities, the air cushion thicknesses measured at the five positions of each group of tests were substituted into the calculation in turn, according to this calculation method, and the results are shown in Table 5 and Figure 7.

From the calculation results, it can be seen that the thickness of the air cushion decreased with the increase in the transverse position coordinate of the tray slot, which was the same as that of the actual use of the air cushion belt conveyor. This calculation method can effectively estimate the thickness of the air cushion to a certain extent. However, it can be seen from Figure 7 that there are still some problems in the calculation results.

First, due to the algorithm’s assumption that the increase in the gas flow rate only exists at the axis of the gas hole, there was a sudden change in the calculation results of the gas flow rate at the axis of the gas hole. The sudden change in the gas flow rate caused the calculation results of the air cushion thickness to undergo abrupt changes; that is, there was a discontinuity of the air cushion value on the air cushion curve. However, in actual situations, the air cushion thickness must be continuous everywhere.

Secondly, the thickness of the air cushion at the lateral center of the tray slot approached infinity. Combining Equation (8) with Figure 6, it could be seen that this was because the lateral change gradient of the air cushion static pressure at this position was 0, and the gas flow rate at this position was considered to be 0.5 times the flow rate of the first exhaust hole, which was a finite value. As a result, the ratio of the two tended to infinity, and the thickness of the air cushion at this position could not be determined.

**Figure 8:**
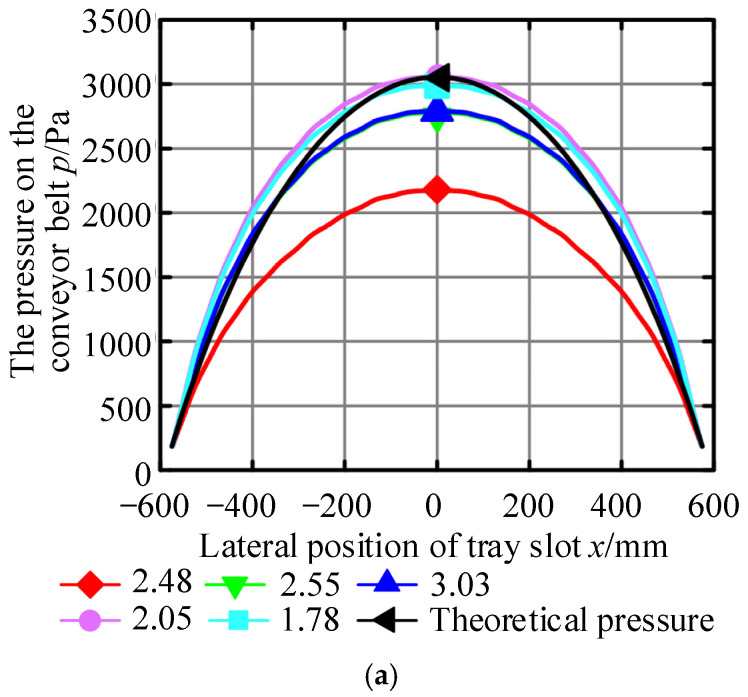

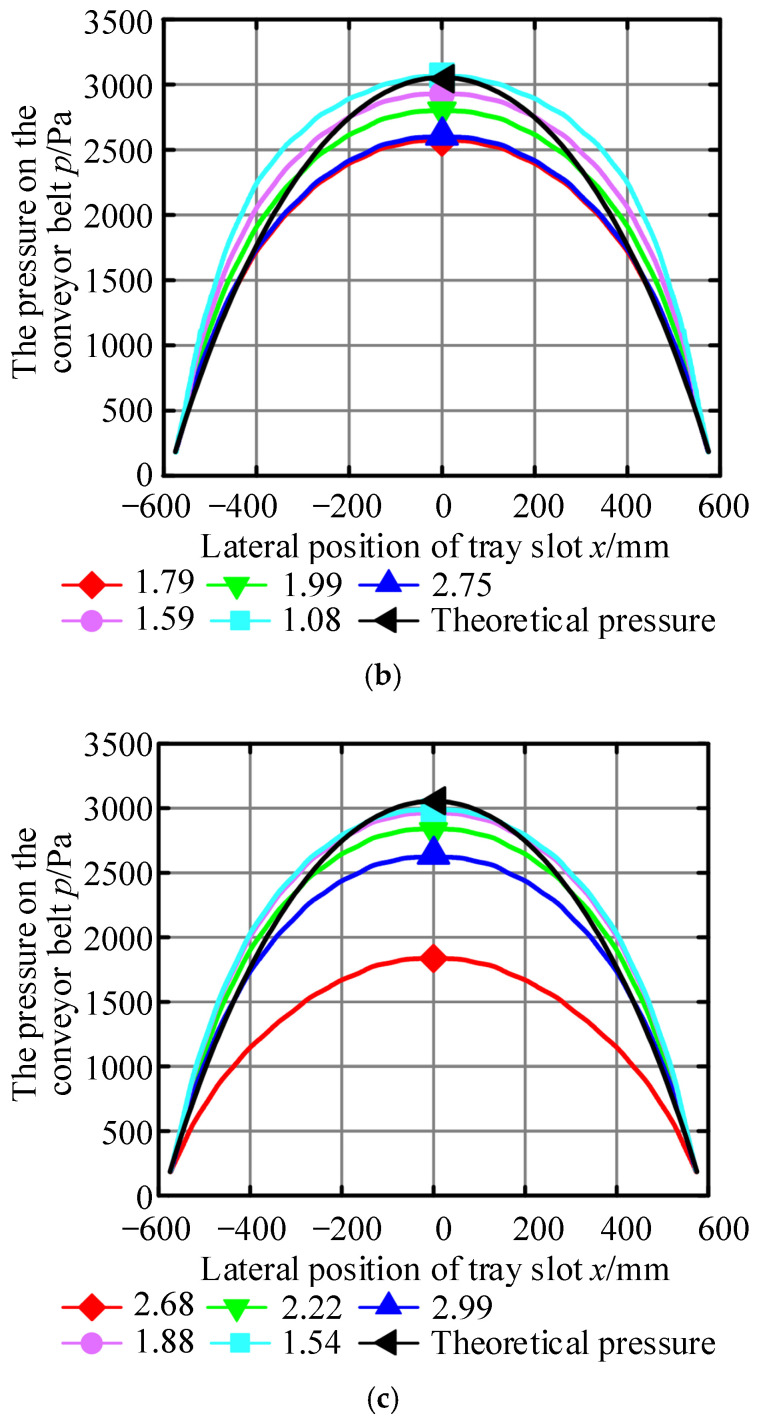
Comparison of simulation and theoretical pressure distribution. (**a**) Pressure Comparison 1; (**b**) Pressure Comparison 2; (**c**) Pressure Comparison 3.

Furthermore, the pressure distribution at the interface between the air cushion and the conveyor belt simulated by Fluent was compared to the theoretical pressure distribution (as shown in Figure 8), and the trend of the two changes showed good consistency.

The experimental, theoretical calculation and the simulation results are summarized in Table 6. It can be seen from the data in Table 6 that when the model of the air cushion flow field was different, different results of the air cushion pressure and the bearing capacity would be obtained by taking the same inlet pressure. Therefore, it can be considered that when the numerical simulation analysis of the air cushion flow field was carried out, the 3D model of the air cushion flow field had a direct influence on the simulation results, and the establishment of the 3D model of the air cushion flow field was based on the calculation results of the air cushion thickness.

Therefore, by further comparing the simulation values with the theoretical and experimental values, and combining them with Table 5, it can be seen that the more accurate the calculation results of air cushion thickness are, the closer the results of the model and simulation are to the theoretical values.

## 4. Improvement of Calculation Method

Regarding the problem found in Section 3.2, that is, since the increased position of the pore flow was considered to exist only at the pore axis during calculation, a sudden change in the flow was obtained at the pore axis during calculation, which caused the calculation result of the air cushion thickness to be sudden and discontinuous. In fact, in the *x* direction, the flow of the air cushion flow field from the left boundary position to the right boundary position of the pore gradually increased as the area of the pore passing through increased, rather than a sudden increase at a certain position. Therefore, the change in flow should be specifically considered during calculation, so as to change the discontinuous calculation result of the air cushion thickness.

When calculating the gas flow rate *Q_xa_* at a certain position *x_a_* in the air cushion flow field, it could be divided into two cases according to the position of *x_a_*:

(1) *x_a_* is between the two exhaust holes; that is $x\in \left(x_{i}+r_{i},x_{i+1}-r_{i+1}\right)$, *x_i_* is the axis position coordinate of the *i*th exhaust hole, *r_i_* represents the radius of the *i*th exhaust hole, and *x_i_*_+1_ is the same as *r_i+_*_1_;

(2) *x_a_* is within a certain pore range; that is $x\in \left[x_{i}-r_{i},x_{i}+r_{i}\right]$.

For case (1), the calculation could be performed according to the original method. For case (2), it was necessary to specifically analyze the pore area *A_xa_* corresponding to the flow rate at the position.

**Figure 9:**
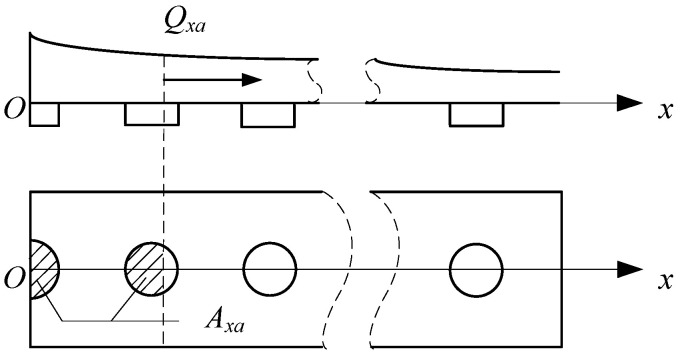
Gas flow at a certain location and its corresponding pore area.

As shown in Figure 9, that shaded portion was the extent of the pores in case (2).

It is not difficult to obtain the equation for calculating *A_xa_* using mathematical methods. Assuming that *x_a_* belongs to the range of the *m*-th exhaust hole, that is, $x_{a}\in \left[x_{m}-r_{m},x_{m}+r_{m}\right]$, then

(10) $$A_{xa}=\sum_{i=1}^{m-1}A_{i}+\arccos\left(\frac{\left|x_{a}-x_{m}\right|}{r_{m}}\right)-\left|x_{a}-x_{m}\right|\sqrt{r_{m}^{2}-{\left(x_{a}-x_{m}\right)}^{2}}$$

In the equation, $\sum_{i=1}^{m-1}A_{i}$ is the sum of the areas of the (*m* − 1) complete pores within the range $\left[0,x_{a}\right]$.

Then, for the gas flow *Q_xa_* at *x_a_*, it can be calculated in two parts, namely

(11) $$Q_{xa}=Q_{xa1}+Q_{xa2}$$

For the interval $\left[0,x_{m}-r_{m}\right]$,

(12) $$Q_{xa1}=Q_{m-1}=\sum_{i=1}^{m-1}CN_{i}A_{i}\sqrt{\frac{2\left(p_{k}-p_{i}\right)}{\rho }}$$

For the interval $\left[x_{m}-r_{m},x_{a}\right]$,

(13) $$Q_{xa2}=\frac{\arccos\left(\frac{\left|x_{a}-x_{m}\right|}{r_{m}}\right)-\left|x_{a}-x_{m}\right|\sqrt{r_{m}^{2}-{\left(x_{a}-x_{m}\right)}^{2}}}{A_{m}}N_{m}q_{m}$$

Substituting Equations (12) and (13) into Equation (11), we finally obtain

(14) $$Q_{xa}=\sum_{i=1}^{m-1}CN_{i}A_{i}\sqrt{\frac{2\left(p_{k}-p_{i}\right)}{\rho }}+\frac{\arccos\left(\frac{\left|x_{a}-x_{m}\right|}{r_{m}}\right)-\left|x_{a}-x_{m}\right|\sqrt{r_{m}^{2}-{\left(x_{a}-x_{m}\right)}^{2}}}{A_{m}}N_{m}q_{m}$$

**Figure 10:**
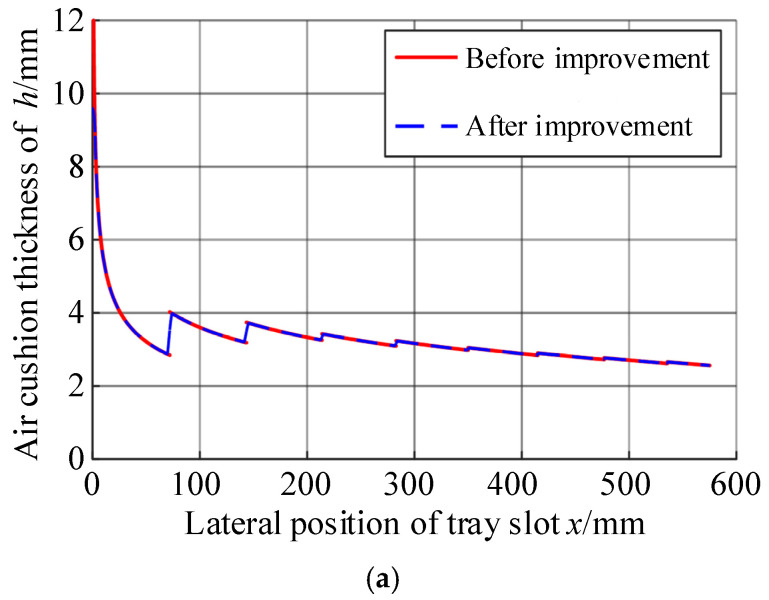

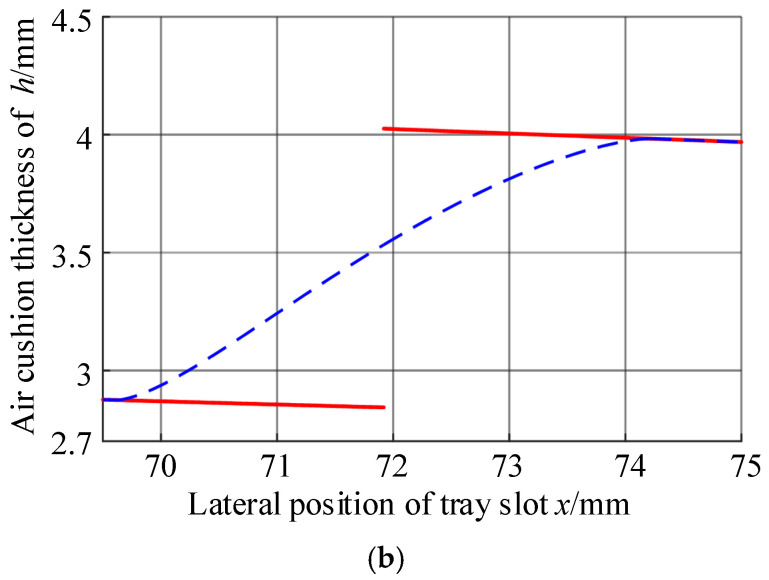
Comparison of results before and after improvement of air cushion thickness calculation method. (**a**) Global distribution; (**b**) local enlargement schematic.

For case (2), the gas flow rate was calculated according to the above equation, and the discontinuity point of the air cushion thickness could be corrected, and the result is shown in Figure 10.

It can be seen that before and after the correction, there were two changes in the calculation results of the air cushion thickness:

(1) At the position *x* = 0, the calculated result of the air cushion thickness no longer tended to infinity;

(2) At *x* = *x_i_*, the air cushion thickness curve was no longer discontinuous, but it changed to increase in a certain trend within the $\left[x_{i}-r_{i},x_{i}+r_{i}\right]$ range.

## 5. Conclusions

Based on the actual situation of the air cushion flow field, reasonable assumptions were made about the properties of the flow field. On the basis of the basic control equations of the fluid mechanics, a mathematical model of the air cushion flow field was established. A flow-based calculation algorithm for calculating the air cushion thickness was derived based on the relationship between the pressure, flow rate, and air cushion thickness in the air cushion flow field. By comparing the experimental data and the simulation results, the calculation algorithm for calculating air cushion thickness was validated. Compared with the experimental test results, the average error of the calculation results of the algorithm proposed in this paper was 14.27%. It was found that the accuracy of the air cushion thickness calculation method based on flow rate was greatly affected by the initial input point. In this paper, the measurement value at 0.5 times the slot angle position was input to obtain a small error, and the calculation result of the air cushion thickness directly affected the accuracy of subsequent 3D model establishment and simulation calculations. However, there were discontinuities in the calculation results of this method at the axis position of the air hole, while the calculation results at the transverse center of the disk groove tended towards infinity. Furthermore, through the analysis of the problems in the calculation method of the air cushion thickness, it is believed that the fundamental reason was the failure to specifically consider the flow rate at the corresponding position, resulting in a sudden change in the gas flow rate at the corresponding position during calculation. Based on this analysis conclusion, mathematical methods were used to supplement and refine the flow calculation at the corresponding location, and the problems existing in the original method were corrected. In the subsequent numerical simulation of the air cushion flow field using Fluent, the calculation of air cushion thickness is a key step in establishing the flow field model, and an improved air cushion thickness calculation method will be used for calculation.

## Figures and Tables

**Table 1:** Bore diameter of each exhaust hole.

Row Number	1	2	3	4	5	6	7	8	9	0
Aperture/mm	4.8	4.6	4.5	3	3	2.2	2.2	2	2	2

**Table 2:** Simulation solver and boundary condition setting.

Solver Type	Pressure-Based Method
Time type	Steady state
Velocity equation	Absolute velocity
Turbulence model	Standard *k* − *ε*
Pressure inlet boundary/Pa	3943/3467/3686
Pressure outlet boundary/Pa	185.1452
Symmetric boundary	Each section of the cut-off section
Solid wall boundary	Tray slot, air hole surface, and interface between air cushion and conveyor belt
Solution method	SIMPLE

**Table 3:** Test results.

Test No.	Air Chamber Pressure/Pa	Air Cushion Thickness at Each Position *h*/mm				
		*x* = −0.509 m	*x* = −0.215 m	*x* = 0.025 m	*x* = 0.263 m	*x* = 0.492 m
1	3943	2.48	2.55	3.03	2.05	1.78
2	3467	1.79	1.99	2.75	1.59	1.08
3	3686	2.68	2.22	2.99	1.88	1.54

**Table 4:** Calculation parameters of air cushion pressure.

Parameter Name	Numerical Value
Conveyor belt unit length gravity *G*_BM_ (N/m)	441.45
Bandwidth *B* (m)	1.8
Tray slot radius *R* (m)	0.878
Material density *γ* (kg/m^3^)	1307.2
Gravitational acceleration *g* (m/s^2^)	9.81
Tray slot angle *λ* (rad)	0.714
Material accumulation angle *β* (rad)	0

**Table 5:** Calculation results of air cushion thickness.

Test No.	Measured Thickness *h*_e_/mm	Calculated Value of Air Cushion Thickness at Each Position *h*/mm					Mean Error/% [*h*_e_ − (*h*_1_ + *h*_2_ + *h*_3_ + *h*_4_ + *h*_5_)/5]/*h*_e_
		*h*_1_ (*x* =−0.503 m)	*h*_2_ (*x* =−0.215 m)	*h*_3_ (*x* =0.025 m)	*h*_4_ (*x* =0.263 m)	*h*_5_ (*x* =0.492 m)	
1	2.48	2.48	3.25	3.88	3.01	2.52	−22.10
	2.55	1.95	2.55	3.04	2.36	1.97	6.90
	3.03	1.91	2.54	3.03	2.35	1.94	22.31
	2.05	1.66	2.22	2.64	2.05	1.69	−0.10
	1.78	1.76	2.3	2.74	2.13	1.78	−20.34
2	1.79	1.79	2.35	2.8	2.17	1.82	−22.12
	1.99	1.52	1.99	2.37	1.84	1.54	6.93
	2.75	1.76	2.31	2.75	2.14	1.79	21.82
	1.59	1.31	1.72	2.05	1.59	1.33	−0.63
	1.08	1.06	1.38	1.63	1.28	1.08	−19.07
3	2.68	2.68	3.51	4.19	3.25	2.72	−22.01
	2.22	1.69	2.22	2.65	2.05	1.72	6.94
	2.99	1.88	2.51	2.99	2.32	1.91	22.34
	1.88	1.53	2.03	2.42	1.88	1.55	−0.11
	1.54	1.52	1.99	2.37	1.84	1.54	−20.26

**Table 6:** Simulation results.

No.	Air Cushion Thickness Substitution Value/mm	Simulation Inlet/Test Chamber Pressure/Pa	Simulation–Theoretical Pressure Distribution Mean Error/Pa	Simulated Bearing Capacity/N	Absolute Error of Bearing Capacity/%
1	2.48	3943	530.751	68.745	−25.155
2	2.55		125.629	89.262	−4.638
3	3.03		122.140	89.591	−4.309
4	2.05		142.763	98.781	4.881
5	1.78		113.993	96.558	2.658
6	1.79	3467	221.200	83.278	−10.622
7	1.99		139.613	91.411	−2.489
8	2.75		208.376	84.066	−9.834
9	1.59		155.875	96.897	2.997
10	1.08		241.840	102.92	9.02
11	2.68	3686	780.831	57.685	−36.215
12	2.22		116.524	91.973	−1.927
13	2.99		195.716	84.484	−9.416
14	1.88		124.464	96.537	2.637
15	1.54		131.936	97.495	3.595

## Data Availability

The data presented in this study are available on request from the corresponding author.
